# The Role and Impact of Deep Learning Methods in Computer-Aided Diagnosis Using Gastrointestinal Endoscopy

**DOI:** 10.3390/diagnostics11040694

**Published:** 2021-04-14

**Authors:** Xuejiao Pang, Zijian Zhao, Ying Weng

**Affiliations:** 1 School of Control Science and Engineering, Shandong University, Jinan 250061, China; 202034876@mail.sdu.edu.cn; 2 School of Computer Science, University of Nottingham, Nottingham NG7 2RD, UK; Ying.Weng@nottingham.edu.cn

**Keywords:** artificial intelligence, computer-aided diagnosis system, deep learning, esophageal lesion, gastric lesion, gastrointestinal endoscopy, intestinal lesion

## Abstract

At present, the application of artificial intelligence (AI) based on deep learning in the medical field has become more extensive and suitable for clinical practice compared with traditional machine learning. The application of traditional machine learning approaches to clinical practice is very challenging because medical data are usually uncharacteristic. However, deep learning methods with self-learning abilities can effectively make use of excellent computing abilities to learn intricate and abstract features. Thus, they are promising for the classification and detection of lesions through gastrointestinal endoscopy using a computer-aided diagnosis (CAD) system based on deep learning. This study aimed to address the research development of a CAD system based on deep learning in order to assist doctors in classifying and detecting lesions in the stomach, intestines, and esophagus. It also summarized the limitations of the current methods and finally presented a prospect for future research.

## 1. Introduction

In recent years, the application of artificial intelligence (AI) based on deep learning in the medical field has become more extensive and suitable for clinical practice compared with traditional machine learning. Constructing a computer-aided diagnosis (CAD) system based on deep learning to assist doctors in diagnosis is of great significance, because diagnosing lesions in the stomach, intestines, and esophagus is laborious for doctors. In addition, misdiagnoses can occur based on a subjective judgment.

### 1.1. Deep Learning

Deep learning is a novel research direction in the field of machine learning. It is based on the self-learning ability to learn complex and abstract features, rather than on traditional machine learning with manual features. Initially, deep learning did not attract much attention from researchers because of hardware limitations; however, it has greatly developed with the continuous progress of computer processing power. Deep learning can help learn the internal regularity and representation levels of training data, and the information obtained is of great help for interpreting the data. Deep learning has led to many achievements in object detection, image segmentation, and classification applications. Compared with traditional machine learning algorithms, deep learning approaches are usually more accurate and robust.

**Figure 1:**
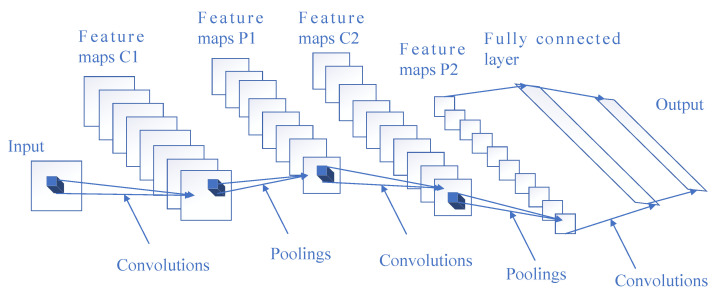
Architecture of a representative convolutional neural network (CNN).

From a technical point of view, deep learning algorithms can make use of a convolutional neural network (CNN) to analyze complex information, which is usually an advantage over manual feature extraction, but it requires learning a great deal of data for accurate inference and analysis. A representative CNN framework is mainly composed of multiple convolutional layers, pooling layers, and fully connected layers, as shown in Figure 1. Convolutional layers are often used for feature extraction. Pooling layers downsample the outputs of convolutional layers, reduce the number of parameters, and speed up calculation. The role of the fully connected layer is to obtain an output by the nonlinear combination of extracted features. The output of the fully connected layers is then input into a softmax activation function to get the final results, so as to generate a prediction of the input data.

### 1.2. Diagnosis of Gastrointestinal Endoscopy Based on Deep Learning

**Figure 2:**
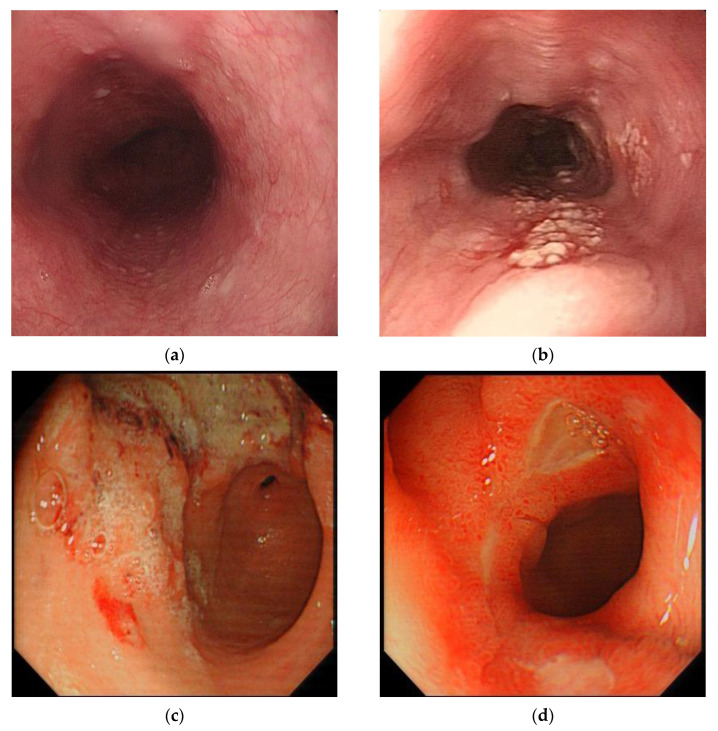
Examples of endoscopic images of normal esophagus, early esophageal cancer (EC), gastric cancer, and duodenal ulcer: (**a**) normal esophagus, (**b**) early EC, (**c**) gastric cancer, and (**d**) duodenal ulcer.

With the continuous progress of deep learning technology, a large number of researchers are paying close attention to its possible application in medical research. This is mainly on account of the characteristics of medical data, which are usually unstructured texts, images, or videos instead of data with distinct characteristics. For example, the endoscopic images of a normal esophagus, early esophageal cancer (EC), gastric cancer, and duodenal ulcer are shown in Figure 2. Therefore, the advancement of deep learning has an inevitable tendency towards medicine. In a CAD system based on traditional machine learning algorithms, researchers manually extract data features, such as the lesion size, edge, and object surface features, based on clinical experience. However, the deep learning algorithm automatically extracts features and learns to recognize them. Using a deep learning algorithm rather than a traditional machine learning algorithm can effectively reduce the loss of feature information, make full use of the feature information for accurate reference, and reduce doctors’ burdens. In recent years, researchers have presented various CAD systems following the success of deep learning in image classification and object detection in medicine. Transfer learning, which transfers information learned in other domains to the current domain, has also been applied to the CAD model because of the shortage of medical data, and has achieved excellent performance.

This study is focused on the research progress of the CAD system based on deep learning to assist doctors in the diagnosis and analysis of gastric, intestinal, and esophageal lesions. The study also presents a concise discussion on deep learning. The main structure of the manuscript is as follows. Section 2.1 addresses the methods of the diagnosis of gastric cancer and *Helicobacter pylori* (HP) infection. Section 2.2 provides the related algorithms based on deep learning to classify and detect colon polyps. The deep learning approaches applied to identify esophageal squamous cell carcinoma (ESCC) and esophageal adenocarcinoma (EAC) in the esophagus are presented in Section 2.3. The manuscript concludes with the summary and the prospect of future research.

## 2. Developments of Deep Learning Methods in CAD for Gastrointestinal Endoscopy

### 2.1. Diagnosis of Gastric Cancer and HP Infection

Gastric cancer is one of the most common types of cancer worldwide; the mortality of gastric cancer is very high. A large number of people die each year from gastric cancer, and about 70% patients with gastric cancer miss the optimal treatment time because their lesions are not found in time [1].

The use of pathological sections is a dependable method to diagnose gastric cancer at present. However, the number of experienced doctors is small, and producing specialized doctor is a time-consuming process. The application of AI technology based on deep learning for object detection and classification has gradually become more popular in the medical field. Ikenoyama et al. [2] compared the diagnostic ability of CNNs with endoscopists for early-stage gastric cancer. They first used 13,584 endoscopic images from 2639 lesions of gastric cancer to train a CNN model based on a single-shot multibox detector (SSD). Then, the performance of this model was compared with 67 endoscopists using a test dataset. After comparing the experimental results between this model and the endoscopists, the proposed CNN model was found to have an obviously higher sensitivity (58.4%) than the experienced endoscopists (31.9%). Moreover, the diagnostic time of this model was significantly shorter compared with that of the endoscopists. Although the performance of the CNN model was not as good as that of the endoscopists in terms of the positive prediction value (PPV) and specificity, it is hoped that the performance will expand through continuous improvement in the model. AI technology based on deep learning can reduce the mortality of patients with gastric cancer, ease the burden of doctors, free them from repeated work, and offer more dependable information. Its results can assist doctors in making an accurate diagnosis and can reduce the number of misdiagnoses and missed diagnosis cases caused by fatigue. It also plays an important role in alleviating doctor–patient conflicts and balancing medical resources between developed and underdeveloped areas.

#### 2.1.1. Diagnosis of Gastric Cancer

In a study that included gastric endoscopic images, Hirasawa et al. [3] proposed a diagnosis system based on deep learning, which was trained by more than 13,000 images of esophagogastroduodenoscopy (EGD), and then evaluated the diagnostic accuracy of this system. The system made use of a deep neural network architecture called SSD, and did not alter its algorithm while using a stochastic gradient descent strategy to fine-tune all of the CNN layers. A duration of 47 s was required by the system to process 2296 test images. This high speed needed to recognize and make inferences about objects cannot be achieved by humans. CNN correctly recognized 71 of 77 gastric cancer lesions with a sensitivity of 92.2%, and 161 noncancerous lesions were detected as gastric cancer, resulting in a PPV of 30.6%. Furthermore, 70 of the 71 lesions (98.6%) with a diameter of 6 mm or more, as well as all invasive cancers, were detected accurately. All missed lesions were superficially depressed and differentiated-type intramucosal cancers were difficult to sift out from gastritis, even for experienced endoscopists. Most false-positive lesions were gastritis with an anomalous mucosal surface or changes in color tone. Their experimental results revealed that the proposed CNN system for detecting gastric cancer could analyze a mass of endoscopic images in a short time with a clinically relevant diagnostic ability. This could be suitable for routine clinical application in order to free endoscopists from their burdensome work. However, here, the problem of a low PPV was not solved.

To overcome the low PPV of the SSD architecture, Sakai et al. [4] proposed a transferring CNN model fine-tuned using the detailed texture information of two kinds of categories—cancer and noncancer. It was capable of showing the proximate locations of early gastric cancers and achieving balanced accuracy regarding the sensitivity and specificity. The experimental results showed that the accuracy, sensitivity, and specificity of their trained model were 87.6%, 80.0%, and 94.8%, respectively, and the correct detection was possible with a high PPV of 93.4%, thus making up for the defect of the SSD network. Although the performance of this model was good, the diagnostic results were prone to errors when the surfaces of the regions were obviously irregular and the target regions were blurry or deep.

**Figure 3:**
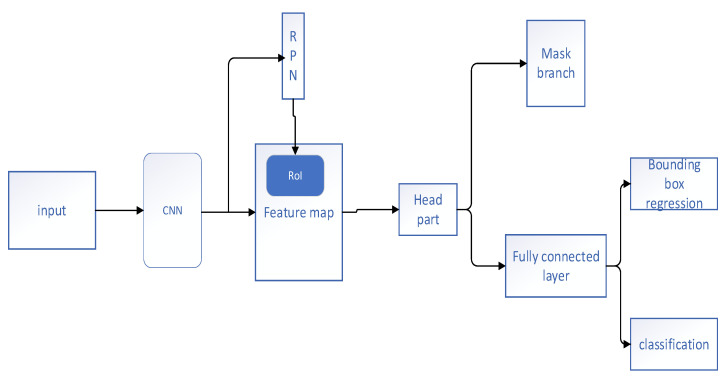
Architecture of the method proposed by Cao et al. [5].

In 2019, Cao et al. [5] developed a mask region-based CNN (Mask R-CNN) method to realize the detection of gastric cancer and for the segmentation of the cancer core. The architecture of the Mask R-CNN used in their study is shown in Figure 3. The model consisted of two parts. In the first part, the whole image was scanned by the basic CNN, generating feature maps that were fed to the region proposal network to generate a region of interest (RoI). Then, the second part classified the RoIs and produced bounding boxes and masks. After proper adjustment, optimization, and data augmentation, this approach could be applied to detect pathological sections of gastric cancer. The experimental results confirmed that this method gained a test result with an average precision (AP) value of 61.2%. Li et al. [6] used a combination of a system based on CNN and a magnifying endoscopy with narrow band imaging (M-NBI) with an outstanding accuracy (90.91%), sensitivity (91.18%), and specificity (90.64%) for diagnosing early gastric cancer. The performance of the specificity and accuracy between the CNN system and experts had little difference; however, the performance of the CNN system was better than those of the nonexperts. The disadvantage of this system was that the images of advanced cancer, polypoid lesions, ulcerated lesions, and low-quality images were excluded during the training process. Hence, this system could not diagnose these diseases, thus limiting its application. In 2020, Shibata et al. [7] also proposed a method using the Mask R-CNN for automatically detecting and segmenting gastric cancer lesions in endoscopic images. They used a residual network (ResNet) as a CNN backbone; the branch mask was made up of seven convolutional layers of a fully convolutional network (FCN). Using a five-fold cross-validation as a performance evaluation, the sensitivity of this model was 96.0%. In the evaluation of the segmentation of the gastric cancer region, the average dice index was 71%. The proposed method was useful for the detection of gastric cancer and for the analysis of the invasive region in the gastrointestinal endoscopy.

**Figure 4:**
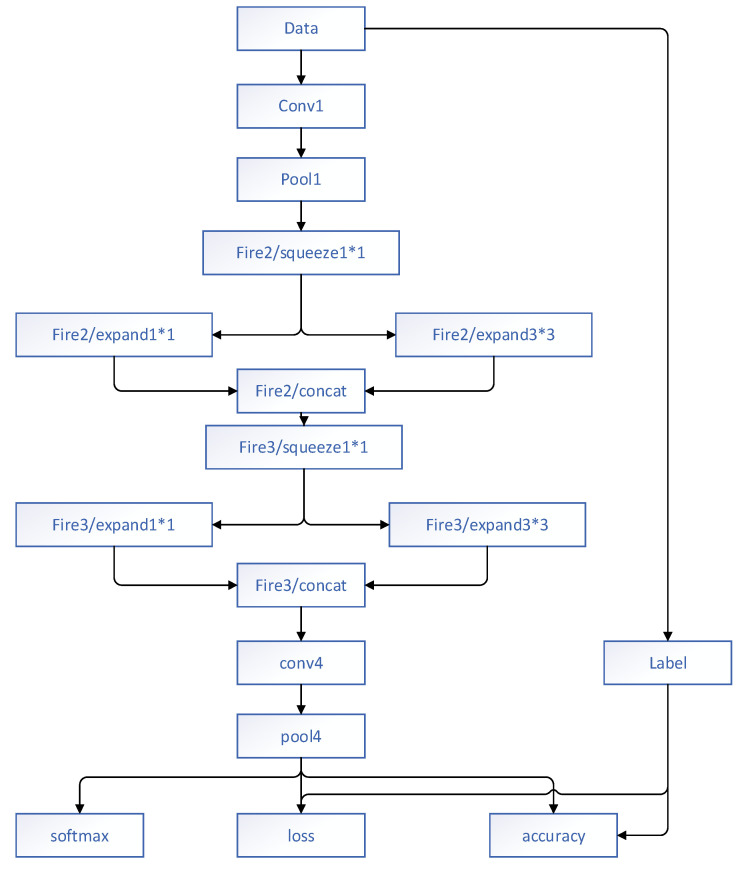
Architecture of the Gastric Precancerous Disease Network (GPD Net) proposed by Zhang et al. [8].

Zhang et al. [8] considered that the models trained from CNNs contained millions of parameters, most of which were likely to cause overfitting in training. To avoid overfitting and achieve quick convergence, they used a concise model called the gastric precancerous disease network (GPD Net) to achieve the classification of three categories of GPD, which were polyp, erosion, and ulcer, respectively. This network introduced fire modules to take the place of traditional convolutional layers. The fire modules were made up of a squeezed convolutional layer (which had only 1 × 1 filters) feeding into an expanded layer (which had both 1 × 1 filters and 3 × 3 filters). The fully connected layers were taken out to realize the FCN. The size and parameters of the model were reduced by approximately 10 times, improving the speed of rapid classification. Moreover, they put forward an innovative fine-tuning method called iterative reinforced learning (IRL) in order to maintain the classification accuracy when training the network with fewer parameters. The experimental results showed that compared with the GPD Net without fire modules, IRL could improve the accuracy by about 9% after six iterations. Finally, the classification accuracy of GPD Net was 88.9%. Therefore, this model could be used with fewer parameters in order to recognize the classification of GPD correctly and to reduce the misdiagnosis rate. The architecture of GPD Net is shown in Figure 4.

#### 2.1.2. Diagnosis of HP Infection

In addition, both Uemura et al. and Tahara et al. found a close relationship between HP infection and gastric cancer [9,10]. Goodwin et al. considered that HP was one of the main causes of HP infection, which causes chronic gastritis, gastroduodenal ulcer, mucosal atrophy, and intestinal metaplasia [11]. Nomura et al. concluded that mucosal atrophy and intestinal metaplasia were known risk factors for gastric cancer [12]. After extensive research, Tahara et al. proposed that the eradication of HP effectively controlled mucosal atrophy and intestinal metaplasia [13]. Therefore, it is of great significance to identify HP infection in order to prevent it from developing further into gastric cancer. Shichijo et al. [14] pretrained a 22-layer CNN on a dataset of 32,208 images, including both positive and negative HP (first CNN), and then fine-tuned it. The images were classified on the basis of eight anatomical positions so as to train another CNN (secondary CNN). The experimental results proved that the diagnostic ability of the CNN was comparable to that of some of the experienced endoscopists, and the performance of the secondary CNN was superior to that of the first CNN. Furthermore, the diagnostic time with the CNN was considerably shorter than that with the endoscopists. The results showed that this recognition system had a sufficient sensitivity and specificity to be introduced into clinical applications; it helped reduce the workload of doctors dramatically.

For establishing an automatic diagnostic system based on deep learning that predicted the HP infection level using gastrointestinal endoscopic images to improve the accuracy and speed of endoscopic examination, Itoh et al. [15] created an effective tool using the CNN that could recognize HP infection. They evaluated the recognition accuracy of the tool with 30 test images by calculating the sensitivity, specificity, and area under the receiver operating characteristic curve (AUC). According to experimental verification, the AUC, sensitivity, and specificity of their CNN for detecting HP infection were 95.6%, 86.7%, and 86.7%, respectively, demonstrating that the CAD system of HP infection appeared to be feasible and might facilitate and improve the diagnosis when applied to clinical practice. In 2018, Nakashima et al. [16] introduced GoogLeNet as a pretrained deep convolutional neural network (DCNN) model, created using a large number of public images in advance. They used data augmentation to increase the number of endoscopic images to fine-tune the GoogLeNet so as to identify the two categories (HP-positive and HP-negative). After experimental verification, the AUC was 66.0%, 96.0%, and 95.0% for white light imaging (WLI), blue laser imaging-bright (BLI-bright), and linked color imaging (LCI), respectively, demonstrating that the developed tool had a better ability to recognize HP infection using BLI-bright and LCI compared with WLI. However, the model was not an ideal model for diagnosing inflammation and other types of gastritis, something the authors hope to improve in further study. The aforementioned methods are summarized in Table 1.

### 2.2. Classification and Detection of Colon Polyps

Gastrointestinal endoscopy plays a pivotal role in the diagnosis and treatment of colorectal cancer (CRC), which is the third leading cause of cancer death in the world [17].

Studies have revealed that most CRCs originate from colon adenomas [18], which are associated with colorectal polyps. Therefore, finding and removing colorectal polyps using endoscopic images is the most effective method to prevent CRC. Nevertheless, small colorectal polyps consist of both adenomatous and nonadenomatous polyps, but the small nonadenomatous polyps are less likely to differentiate into CRCs [19]. Therefore, it is significant to avoid the needless removal of colorectal polyps by distinguishing adenomatous polyps from nonadenomatous polyps. Although an accurate identification of adenomatous and nonadenomatous polyps through endoscopic findings is very important in order to avoid the redundant resection of colorectal polyps, diagnosing small colorectal polyps accurately is difficult even for experienced doctors at times [20]. Consequently, it is necessary to develop a precise and dependable CAD system to discriminate adenomatous and nonadenomatous polyps in endoscopic images.

The difficulty in detecting colorectal polyps automatically is not only the variation inside colorectal polyps, but also the small difference between normal mucosa and polyps, which is the bottleneck of the manual extraction method. In contrast, deep learning methods can extract more detailed information and features of polyps from endoscopy images at a pixel level through end-to-end learning. A large number of research outcomes have demonstrated that the polyp feature information extracted by the deep learning method is markedly better than that extracted by other hand-crafted extraction methods, because it can be better suited to the intricate clinical environment and is more suitable for the automatic detection and classification of colorectal polyps in clinical practice.

**Figure 5:**
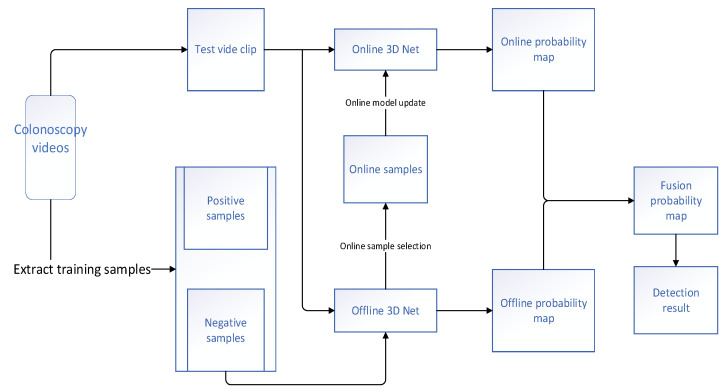
Flowchart of the online and offline 3D model proposed by Yu et al. [22].

**Figure 6:**
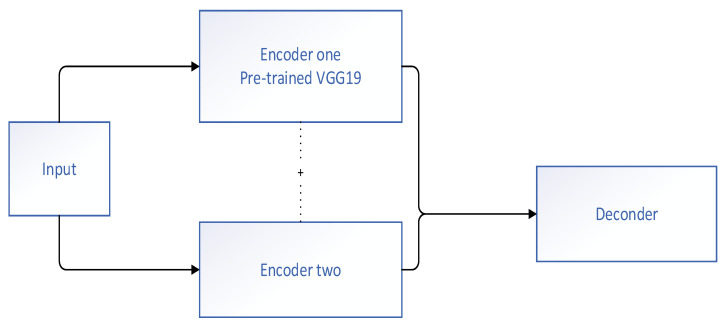
Main framework of Y-Net proposed by Mohammed et al. [23].

Tajbakhsh et al. [21] constructed a novel CAD polyp detection system from endoscopic videos in 2015. This method did not rely on one or only a subset of polyp characteristics; rather, it fully used all the obtainable image features containing color, texture, shape, and temporal information. Initially, geometric features, such as the shape and size of the polyps, were used to obtain a set of candidate regions of existing polyps. Then, a combination of CNNs was used to classify the interesting regions. The outputs of the combined CNN were averaged to acquire a probabilistic map of the presence of polyps in the frame. The experimental findings demonstrated that this system significantly improved the performance of detection among the other methods and reduced the number of false positives. Yu et al. [22] presented an innovative offline and online three-dimensional (3D) deep learning integrated network. The flowchart of the proposed network is shown in Figure 5. They leveraged the 3D model to decrease the number of false positives and further enhanced the discriminant ability of the network for a specific video. A large number of experiments showed that the 3D model was able to automatically learn more typical temporal and spatial feature information from colonoscopy videos, and thus had more significant discriminant ability compared with the previous approaches using manual features or two-dimensional (2D) CNN. Although the model provided good diagnostic results in distinguishing hard mimics, it was still difficult to identify some particularly similar regions. The authors of the model believed that the problem could be improved by manually annotating these similar regions and putting them into the training dataset to train the model. Mohammed et al. [23] also came up with a novel deep learning model called Y-Net that contained two encoder architectures and one decoder architecture to implement the detection of automatic polyps (Figure 6). The presented Y-Net method depended on the efficient use of pretrained and un-pretrained models with a novel sum-skip-concatenation strategy. Each of the encoders was trained by an encoder-specific learning rate along the decoder. Compared with the early models that used hand-crafted features or 2D/3D CNN, the presented method was superior to the pre-existing models in the performance of polyp detection. The model was validated on the Arizona State University and Mayo Clinic (ASU-Mayo Clinic) polyp database of Medical Image Computing and Computer Assisted Intervention (MICCAI) 2015 Challenge for polyp detection, and the performances of the F1-score, F2-score, and precision rate were 85.9%, 85.0%, and 87.4%, respectively. However, still, some failure examples (false-positives and false-negatives) were detected in the diagnostic results of their model; although the changes in light and contrast were taken into consideration, no significant improvement in accuracy was found. In 2020, Haj-Manouchehri et al. [24] used the neural network to achieve the detection and segmentation of polyps in frames. In the polyp detection part, they proposed an innovative CNN based on the VGG network, and achieved an accuracy of 86.0% on the newly collected dataset. In the polyp segmentation part, a valid post-processing algorithm using an FCN was proposed. They verified the constructed polyp segmentation model on the ETIS-LARIB database and realized a final F2 score of 82.0%, indicating that the performance of this method was superior to that of the methods that took part in the subchallenge of MICCAI.

Training the CNN is difficult because of the tendency of labeled datasets in the medical domain to be small. Employing a transfer learning method is an alternative approach, by making full use of the learned information from other domains’ large datasets to deal with this problem [25]. When a moderate dataset size for the target source is obtained, fine-tuning of the CNN can be implemented [26]. Nevertheless, if the number of images of the target source is small, it is also useful to directly transfer CNN features from the original source without fine-tuning [27]. Therefore, in 2017, Zhang et al. [28] proposed a new strategy for CRC diagnosis by transferring low-level features learned from all kinds of nonmedical domains into the current task using a deep CNN. Initially, the polyp image was identified from the nonpolyp image and was then predicted. The experimental results indicated that the proposed automated CRC diagnosis strategy had a similar accuracy in terms of minimal preprocessing procedures compared with visual inspection by endoscopists and early state-of-the-art approaches, but had a better recall rate and accuracy. Therefore, the system assisted endoscopists in identifying the omissive adenomatous polyps for timely resection. The disadvantage of this system was that it failed to further study the structure of CNN to directly classify polyps (support vector machine is used), which will also be one of the research directions of the authors of the system in the future. The aforementioned methods are concluded in Table 2.

### 2.3. Diagnosis of ESCC and EAC in Esophagus

EC is one of the most common types of malignant cancer and was the sixth leading cause of cancer death worldwide in 2018 [17]. EC has three histological types: ESCC, EAC, and undifferentiated carcinoma, among which ESCC is the most common. The CNN-based diagnostic method has been applied in a few studies to improve the accuracy of esophageal lesions detected using endoscopy. Diagnostic methods based on CNN have been employed in a few studies. In 2019, Horie et al. [29] developed a diagnostic system based on deep learning, which was constructed on a variety of EGD images with a satisfying efficiency (0.02 s for one image), in order to achieve the detection of EC consisting of squamous cell carcinoma and adenocarcinoma. This showed that this system was promising for achieving a real-time diagnosis. This system employed a deep neural network model called SSD, which was a deep CNN composed of 16 layers or more, without altering its architecture. All of the layers of the model were fine-tuned using stochastic gradient descent. This strategy demonstrated that the sensitivity of CNN diagnosis for each case was high. Moreover, all seven lesions less than 10 mm in size could be detected by this system.

#### 2.3.1. Diagnosis of ESCC

In 2019, Cai et al. [30] proposed an identification system based on images from conventional endoscopy with standard white light called Deep Neural Network-Computer-Aided Detection (DNN-CAD) in order to detect inchoate ESCC. They augmented the training data by 10 times by cropping the images that were collected, so as to improve the performance of this system. Finally, they annotated 187 white light images of the esophagus to validate the proposed DNN-CAD system with an accuracy of 91.4%, a sensitivity of 97.8%, and a specificity of 85.4%. Hence, it was concluded that this system achieved an excellent diagnostic ability to diagnose early-stage ESCC. Furthermore, the system also output the bounding box of latent cancer lesions, thus sending a reminder to the doctor to focus on the suspicious lesion. They intended to improve the performance of the system to identify the histologic characteristics of EC so as to achieve further classification. A year later, Guo et al. [31] used a deep learning model based on the SegNet framework that was a deep encoder–decoder network for various categories of pixelwise segmentation. For both endoscopic images and video datasets, this model showed a better sensitivity (98.04%) and specificity (95.03%). Obviously, the CAD system appeared to provide capacity in diagnosing the accurate locations of esophageal precancerous lesions and early-stage ESCC with appropriate training datasets and skills. However, randomized controlled trials should be designed to further verify the practical applicability of this model. Ohmori et al. [32] also constructed a computer-aided image analysis system based on deep learning to detect and distinguish ESCC. They used SSD as the basic model and fine-tuned all layers of the model through the backpropagation algorithm. Their system creatively combined WLI, narrow band imaging (NBI)/blue laser imaging (BLI), non-magnified endoscopy (non-ME), and magnified endoscopy (ME) to detect and distinguish ESCC. Their experimental results showed an excellent performance for detecting ESCC, and had a prominent ability to differentiate ESCC from noncancerous lesions and normal mucosa. After constant research, Tokai et al. [33] found that the treatment varied according to the invasion depth of ESCC. Therefore, the diagnosis of the invasive depth of ESCC is of great significance before effective treatment. The authors of [33] constructed a CAD system to detect the invasion depth of ESCC. First, they used SSD as a basic architecture to detect the superficial ESCC from the endoscopic images. Then, GoogLeNet was used to diagnose the invasion depth of ESCC. After experimental verification, it was concluded that the AI-diagnostic system demonstrated a higher diagnostic accuracy for the ESCC invasion depth compared with that of endoscopists. Therefore, it is very helpful for treating ESCC.

#### 2.3.2. Diagnosis of EAC

**Figure 7:**
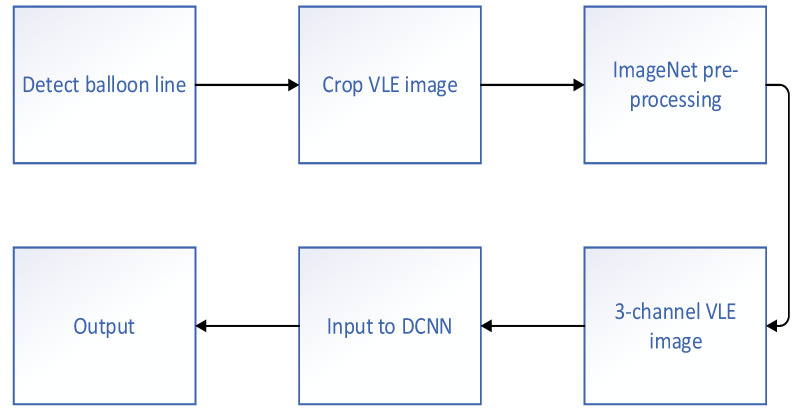
Framework of the method proposed by Fonollà et al. [38].

ESCC still has the highest incidence. However, EAC incidence has been growing promptly all over the world in the last few years, and is expected to realize further growth [34]. Barrett’s esophagus (BE) is a risk factor for the development of EAC. When BE is related to dysplasia, the risk of EAC increases [35]. However, the ability of endoscopic surveillance for BE has been disappointing. A CAD system based on deep learning with the CNN can help endoscopists in detecting Barrett’s dysplasia. Mendel et al. [36] studied the diagnosis of BE using CNN based on deep ResNet to identify patches in a high-definition white light endoscopy (HD-WLE) image as cancerous and noncancerous. The image was first divided into 224 × 224 patches without overlapping and was then categorized into cancerous and noncancerous based on a specific threshold (*t*). Each patch that had an output probability was compared with *t* to decide whether it was a noncancerous region. The performance of this system was evaluated by leave-one-patient-out cross-validation and finally realized a sensitivity and specificity of 94.0% and 88.0%, respectively. Compared with other systems based on the same dataset, the performance of this system was superior to that of others. Hashimoto et al. [37] designed a CNN model to perform the detection of early esophageal neoplasia in BE. Their architecture comprised two steps. The first was a binary classification based on Xception architecture to rapidly flag interesting frames, and the second step was to find the localization of the lesion through you only look once (YOLO) v2 on positively recognized frames from the first step. The performance of the binary classification (dysplasia vs. no dysplasia) method showed a satisfying sensitivity (96.4%), specificity (94.2%), and accuracy (95.4%) per image on an internal validation dataset. The localization method also accurately detected a large proportion of lesions with a mean AP of 75.3%, sensitivity of 95.6%, and positive predictive value of 89.2%. A major advantage was that the model was quick enough to realize real-time detection and diagnosis. Fonollà et al. [38] also discovered that a brief but deep model was the most promising strategy to identify a volumetric laser endomicroscopy (VLE) RoI between nondysplastic BE (NDBE) and high-grade dysplasia (HGD) because of the unbalanced property of the dataset. Thus, they chose to use a combination of horizontal flip, motion blur, and optical grid distortion of three DCNNs, each of them based on the VGG16 network, in order to find neoplasia using a valid VLE image dataset. On the basis of the VGG16 network, they appended a global average pooling layer after the final convolutional layer, which was effective in reducing the number of parameters from 7 × 7 × 512 to 1 × 1 × 512 in the model. The framework of the proposed network is shown in Figure 7. In the end, their model acquired a specificity of 85%, a sensitivity of 95%, and an AUC of 96% on the test dataset with the multi-frame analysis, which obviously surpassed the early works. Ghatwary et al. [39] found that a few advanced object detection approaches using CNN, including region-based CNN (R-CNN), fast R-CNN, faster R-CNN, and SSD, were adapted to automatically detect abnormal regions in HD-WLE images of the esophagus. Therefore, they employed VGG16 as the basic architecture to extract feature information and to compare the performance among the aforementioned approaches. They conducted extensive evaluation experiments in terms of different evaluation indicators. The results revealed that the SSD model outperformed other approaches, with a remarkable outcome of 96.0% (sensitivity), 92.0% (specificity), and 94.0% (F-measure) when assessed based on five-fold cross-validation. This research was an important step forward to use deep learning object detection algorithms to detect abnormalities in esophageal endoscopy images. The aforementioned methods are summarized in Table 3.

## 3. Discussion

Gastrointestinal endoscopy plays a very important role in the diagnosis and treatment of gastrointestinal diseases. More doctors are needed who can perform accurate diagnosis and treatment because of the increasing incidence of gastrointestinal diseases in recent years. A CAD system based on the deep learning algorithm has been explored by a large number of researchers in order to reduce the burden of doctors and to improve the accuracy of diagnosing lesions in the stomach, intestines, and esophagus.

This study reviewed the research progress of the CAD system based on deep learning for assisting doctors with classifying and detecting gastric, intestinal, and esophageal diseases. As observed in Table 1, Table 2 and Table 3, many researchers built CAD systems with a good performance based on the CNN applied to extract feature information using convolution layers, down-sampled the feature maps by pooling layers, and obtained the output by nonlinear combination of extracted features through the fully connected layers. On the one hand, some researchers used the transfer learning method based on a basic network to train the network with a smaller dataset. The basic CNN models usually included VGG, GoogLeNet, ResNet, SSD, Mask R-CNN, and so forth. The method of transfer learning to construct a CAD system could make use of the knowledge acquired by the model with a large number of training data, so as to effectively alleviate the problem of overfitting caused by the lack of training data. On the other hand, researchers have improved the structure of that network, by creatively putting forward novel network structures combined with pre-processing and post-processing technology. Creatively putting forward a new network structure is a link between the past and the future. The pre-processing and post-processing technology can effectively improve the performance of the network. The two aforementioned strategies can make the model achieve an excellent performance; the performance of the model using the transfer learning strategy is usually better than that of the model trained directly with the target dataset. Moreover, in the process of model training, the model trained with a transfer learning strategy uses less data than the model trained directly with the target dataset. If prior knowledge can be further applied to the new network structure, it is expected to improve the performance of the model. Moreover, by summarizing the aforementioned methods, the imaging model has been found to have a significant impact on the diagnostic performance of the CAD system. Thus, we believe that using multimodal images as a mixed input can provide more information, which will be of great help to improve the diagnostic performance of the CAD system.

Compared with the traditional machine learning methods, CAD systems based on deep learning can effectively improve diagnosis performance and reduce the rate of misdiagnosis and missed diagnosis, while reducing the burden on doctors. It is of great significance to help doctors carry out a remote diagnosis for improving the level of medical diagnosis in remote areas. However, it is difficult to understand the inner workings of CAD systems based on deep learning because the feature activities of each layer are usually complex and abstract to fully understand [40,41], which is also called the black-box problem. Therefore, the CNN gains a better generalization ability at the expense of its interpretability. Even if the results are correct, doctors still cannot fully trust the CAD system. As a result, whether the CAD system can replace doctors in diagnosis is still controversial.

In addition, by summarizing the current research methods, we find that besides the unique shortcomings of the current model, some common limitations still need to be resolved. First of all, training an accurate neural network needs a lot of data. However, it is difficult to obtain and label large amounts of data in the medical field, which makes the trained model prone to overfitting. Second, most training data come from a single research center or hospital; hence, the designed model can only perform well on a specific dataset, but performs poorly on other datasets. Third, most of the current CAD systems depend on high-quality images to make a diagnosis. The performance of the systems using low-quality images (such as blurry or low-resolution images) for training cannot be determined. Fourth, the neural network contains a large number of parameters, and hence it is time-consuming to diagnose the corresponding diseases. Thus, it is difficult to meet the real-time requirements of clinical practice, especially on a 3D model. Fifth, the current CAD system is usually based on the 2D CNN of 2D images to diagnose gastrointestinal endoscopic lesions. However, the information provided by 2D images is limited, and sometimes it cannot fully represent the lesions. In contrast, the 3D CNN model based on videos or 3D images can make full use of temporal and spatial information. Therefore, much work still needs to be done on the research of 3D CAD systems. Furthermore, each of the aforementioned models is only effective for a specific disease and fails to realize information interaction between diseases, thus limiting the application of the models. Finally, although researchers have developed a variety of systems, the performance of these models cannot be uniformly compared because of the different datasets and evaluation criteria used in each system. Thus, the lack of a unified dataset and common criteria for verification is also detrimental to the development of CAD systems.

## 4. Conclusions

In conclusion, a CAD system based on the deep learning method can automatically extract and recognize features. Using the deep learning method rather than traditional machine learning can effectively reduce the omission of feature information and make full use of the feature information to improve the performance of diagnosing lesions in the stomach, intestines, and esophagus. It can also assist endoscopists through auxiliary advice and reducing their burden. Moreover, it is of great help to solve the problem of insufficient medical resources in remote areas by employing an online CAD system.

We think that it is necessary to make improvements in the following aspects in order to promote the further development of CAD systems. Initially, the dataset for training needs to be augmented as much as possible and needs to be collected from a wide range of sources in order to obtain a network model with a better generalization ability. Then, to deal with the shortage of training data, both weakly supervised learning methods and transfer learning methods are excellent for avoiding the annotation of a large amount of data. We intend to use a few-shot learning strategy to achieve excellent diagnostic accuracy based on a small amount of labeled data in the following research. Third, some image preprocessing techniques, such as image deblurring and resolution improvement, can be used to improve the performance of the model when low-quality images are input. We still need to make further improvements on 3D CAD systems in order to acquire more feature information, and should implement further speed optimization to achieve real-time clinical applications while ensuring excellent performance, especially on 3D models. Furthermore, when something goes wrong in one part of the body, it often sets off alarms elsewhere because the body is a collaborative group of organs. Therefore, when constructing a CAD system, we should pay more attention to the transfer of the feature information of different diseases, try to find the relationship among different diseases, and establish a CAD system that can diagnose multiple related diseases. More importantly, the trust between CAD systems and doctors should be built by increasing the interpretability of CNN. Last, but not least, a unified validation dataset and common criteria are needed to make accurate comparisons among various CAD systems so as to facilitate further development.

## Figures and Tables

**Table 1:** Characteristics of models for the diagnosis of gastric cancer and *Helicobacter pylori* (HP) infection.

Study	Aim	Method	Performance	Train Dataset	Test Dataset
Ikenoyama et al. (2021) [2]	Comparison between CNN and endoscopists	CNN based on SSD	CNN/Endoscopist: Sensitivity: 58.4%/31.9% Specificity: 87.3%/97.2% PPV: 26.0%/46.2%	10,474 early-stage gastric cancer images and 3110 advanced-stage gastric cancer images	209 gastric cancer images and 2731 normal images
Hirasawa et al. (2018) [3]	Detection	CNN based on SSD	Sensitivity: 92.2% Accuracy: 98.6% PPV: 30.6%	13,584 gastric cancer images	2296 gastric cancer images
Sakai et al. (2018) [4]	Detection	CNN based on GoogLeNet	Accuracy: 87.6% Sensitivity: 80.0% Specificity: 94.8%	9587 gastric cancer images and 9800 normal images	4653 gastric cancer images and 4997 normal images
Cao et al. (2019) [5]	Detection + segmentation	Mask R-CNN	AP: 61.2%	1000 positive samples and 250 negative samples	120 positive samples and 29 negative samples
Li et al. (2020) [6]	Classification	CNN + M-NBI	Accuracy: 90.91% Sensitivity: 91.18% Specificity: 90.64%	1702 gastric cancer images and 386 normal images	170 gastric cancer images and 171 normal images
Shibata et al. (2020) [7]	Detection	Mask R-CNN	Average Dice: 71.0% Sensitivity: 96.0%	533 gastric cancer images and 1208 normal images	Five-fold cross-validation
Zhang et al. (2017) [8]	Classification	GPD Net	Accuracy: 88.9%	921 images of erosion, 918 images of polyps, and 944 images of ulcer	300 images of erosion, 300 images of polyps, and 300 images of ulcer
Shichijo et al. (2017) [14]	Classification	First CNN based on GoogLeNet Secondary CNN based on GoogLeNet	First/second AUC: 83.1%/87.7% Sensitivity: 81.9%/88.9% Specificity: 83.4%/87.4%	32,208 images either positive or negative for HP	11,481 images
Itoh et al. (2018) [15]	Detection	CNN based on GoogLeNet	AUC: 95.6% Sensitivity: 86.7% Specificity: 86.7%	596 images	30 images
Nakashima et al. (2018) [16]	Classification	CNN based on GoogLeNet	AUCs: 66.0% (WLI), 96.0% (BLI-bright), 95.0% (LCI)	648 images for each WLI, BLI-bright, and LCI	60 separate images for WLI, BLI-bright, and LCI

**Table 2:** Characteristics of models for the classification and detection of colon polyps.

Study	Aim	Method	Performance	Train Dataset	Test Dataset
Tajbakhsh et al. (2015) [21]	Detection	Three-way image presentation + CNN	Sensitivity: about 75.0%	20 collected short colonoscopy videos (10 positive, 10 negative)	20 collected colonoscopy videos (10 positive and 10 negative)
Yu et al. (2017) [22]	Detection	Offline and Online 3D FCN	F1-score: 78.6% F2-score: 73.9% Precision: 88.1% Recall rate: 71.0%	ASU-Mayo clinic database (20 colonoscopy videos)	ASU-Mayo clinic database (18 short colonoscopy videos)
Mohammed et al. (2018) [23]	Detection	Y-Net	F1-score: 85.9% F2-score: 85.0% Precision: 87.4% Recall rate: 84.4%	ASU-Mayo clinic database (20 colonoscopy videos)	ASU-Mayo clinic database (18 short colonoscopy videos)
Haj-Manouchehri et al. (2020) [24]	Detection + segmentation	CNN based on VGG; FCN + post-processing	Detection: (accuracy) 86.0% Segmentation: (F2-score) 82.0%	Two collected colonoscopy videos for detection; CVC-CLINIC and ETIS-LARIB dataset for segmentation	1 collected colonoscopy video for detection; CVC-CLINIC and ETIS-LARIB datasets for segmentation
Zhang et al. (2017) [28]	Detection + classification	CNN based on CaffNet	Precision: 87.3% Recall rate: 87.6% Accuracy: 85.9%	Source: ImageNet database and Places205; Target: PWH database and new polyp database	50 images for each class (nonpolyp, hyperplasia, and adenoma polyps) + 10 images for each class (hyperplasia, serrated adenoma, and adenoma) for 5 trials

**Table 3:** Characteristics of models for the diagnosis of esophageal squamous cell carcinoma (ESCC) and esophageal adenocarcinoma (EAC) in the esophagus.

Study	Aim	Method	Performance	Train Dataset	Test Dataset
Horie et al. (2019) [29]	Detection of ESCC + EAC	CNN based on SSD	Sensitivity: 97.0% (ESCC) 100.0% (EAC) for each case	8428 EC images	1118 images (EC + normal)
Cai et al. (2019) [30]	Detection of ESCC	DNN-CAD	Accuracy: 91.4% Sensitivity: 97.8% Specificity: 85.4%	2428 (1332 abnormal and 1096 normal) esophagoscopic images	187 images
Guo et al. (2020) [31]	Detection of ESCC	CNN based on SegNet	AUC: 98.9% Sensitivity: 98.04% Specificity: 95.03%	2770 images (precancerous and early-stage ESCC); 3703 images (noncancerous)	Dataset A: 1480 images (precancerous + ESCC); B: 5191 images (noncancerous); C: 27 videos (precancerous + ESCC); D: 33 videos (noncancerous)
Ohmori et al. (2020) [32]	Detection of ESCC	CNN based on SSD	Performance is good; 100.0% sensitivity by non-ME and 98.0% by ME	9591 non-ME + 7844 ME images from superficial ESCCs; 564 non-ME + 2744 ME images of noncancerous lesions; 1128 non-ME + 691 ME images of normal esophagus	255 non-ME WLI images; 268 non-ME NBI/BLI images; 204 ME NBI/BLI images
Tokai et al. (2020) [33]	Invasion depth of ESCC	CNN based on SSD (detection), GoogLeNet (estimation)	Detection: 95.5% Estimation of depth: 84.1% (sensitivity) 80.9% (accuracy)	1751 images of ESCC	291 test images
Mendel et al. (2017) [36]	Diagnosis of EAC	CNN based on ResNet	Sensitivity: 94.0% Specificity: 88.0%	4157 noncancerous region patches and 3666 cancerous region patches	Leave-one-patient-out cross-validation
Hashimoto et al. (2020) [37]	Detection of EAC	Model based on Xception and YOLO v2	Accuracy: 95.4% Sensitivity: 96.4% Specificity: 94.2%	916 images of BE (high-grade dysplasia/T1 cancer) and 919 images of BE (nonhigh-grade dysplasia)	458 test images (225 dysplasia and 233 non-dysplasia)
Fonollà et al. (2019) [38]	Diagnosis of EAC	Assemble of 3 DCNN based on VGG16	AUC: 96.0% Sensitivity: 95.0% Specificity: 85.0%	134 NDBE, 38 HGD/EAC regions; total 8772 images	99 NDBE and 42 HGD/EAC; total of 7191 images
Ghatwary et al. (2019) [39]	Comparison	CNN based on R-CNN/Fast R-CNN/Faster R-CNN/SSD	SSD is the best: Sensitivity: 96.0% Specificity: 92.0% F-measure: 94.0%	50 images (EAC) and 50 images (noncancerous) before data augmentation	Leave-one-patient-out cross-validation
